# Molecular regulation of autophagosome formation

**DOI:** 10.1042/BST20210819

**Published:** 2022-01-25

**Authors:** Yan Hu, Fulvio Reggiori

**Affiliations:** Department of Biomedical Sciences of Cells and Systems, Molecular Cell Biology Section, University of Groningen, University Medical Center Groningen, Groningen, The Netherlands

**Keywords:** ATG proteins, autophagy, degradation, lysosomes, phagophore, sequestration

## Abstract

Macroautophagy, hereafter autophagy, is a degradative process conserved among eukaryotes, which is essential to maintain cellular homeostasis. Defects in autophagy lead to numerous human diseases, including various types of cancer and neurodegenerative disorders. The hallmark of autophagy is the *de novo* formation of autophagosomes, which are double-membrane vesicles that sequester and deliver cytoplasmic materials to lysosomes/vacuoles for degradation. The mechanism of autophagosome biogenesis entered a molecular era with the identification of autophagy-related (ATG) proteins. Although there are many unanswered questions and aspects that have raised some controversies, enormous advances have been done in our understanding of the process of autophagy in recent years. In this review, we describe the current knowledge about the molecular regulation of autophagosome formation, with a particular focus on budding yeast and mammalian cells.

## Introduction

**Figure 1.**
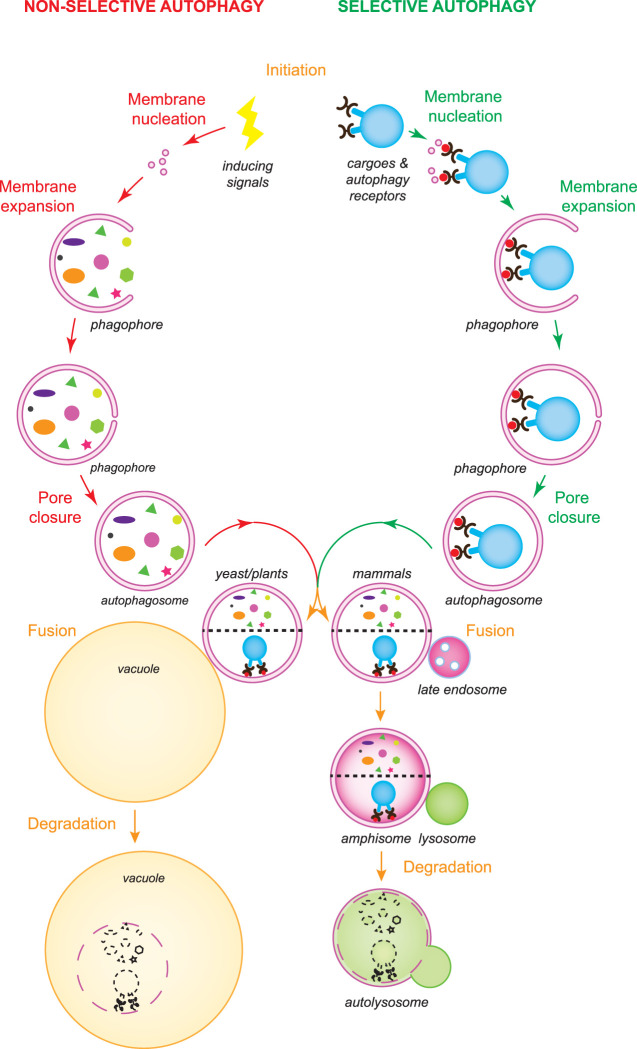
Overview of the autophagy process. There are two types of autophagy: non-selective and selective autophagy. While non-selective bulk autophagy is induced by stress and other signals, selective autophagy is triggered by cargoes that must be turned over and autophagy receptors (black pincers) are central in this induction event. The fusion of vesicles, probably of different origins, leads to the phagophore nucleation. Subsequently, the expansion of the phagophore by the acquisition of extra lipids and the closure of the final pore lead to the formation of an autophagosome. The intracellular material that has to be recycled is sequestered during autophagosome biogenesis. By interacting with the Atg8/LC3-PE pool (red circles) in the inner surface of the expanding phagophore, the autophagosome receptors guide the exclusive sequestration of the cargoes targeted to degradation. Complete autophagosomes fuse with vacuoles in yeast and plants, and with lysosomes in mammals, delivering their cargo into the acidic lumen of these organelles. Autophagosomes can also fuse with late endosomes in plants and mammals, before fusing with vacuoles and lysosomes, respectively. Vacuolar and lysosomal resident hydrolases degrade the delivered cargoes into basic metabolites, which are then transported into the cytoplasm for recycling.

Autophagy is a self-degradative cellular process conserved among eukaryotes, which is involved in multiple physiological functions such as the turnover of cellular materials and organelles to maintain cellular homeostasis for example to adapt to stresses and developmental programs [1]. Dysfunctional autophagy has been connected with several human diseases, including cancers, pathogen infection, diabetes and neurodegenerative disorders [2]. It was first discovered at the end of the 1950s in mammalian cells [3] and further deciphered in the 1990s, when the *autophagy-related* (*ATG*) genes were identified and gradually decrypted in *Saccharomyces cerevisiae* [4]. During autophagy, intracellular materials are sequestered into double-membrane vesicles termed autophagosomes that are formed *de novo*, and then delivered into mammalian lysosomes or yeast and plant vacuoles for turnover. Depending on the sequestration mechanism, autophagy is divided into non-selective and selective. While cytoplasmic components are engulfed in an apparent random manner by autophagosomes during non-selective bulk autophagy, the so-called autophagy receptors are central in recognizing the cargoes that are specifically targeted for degradation via selective types of autophagy (Figure 1), which among others include mitophagy, pexophagy, ER-phagy and aggrephagy [5].

The hallmark of autophagy is the biogenesis of an autophagosome. This event is initiated upon either autophagy-inducing signals or binding of the autophagy receptors to the targeted cargoes, and it is followed by the nucleation of a small cytoplasmic membranous cisterna known as phagophore or isolation membrane (Figure 1), which is generated at a specific location called the phagophore assembly sites (PAS) in yeast or omegasomes in mammalian cells [6,7]. The phagophore subsequently expands and closes into an autophagosome (Figure 1). Autophagosome biogenesis relies on the function of the ATG proteins and approximately 20 of them are part of the core ATG machinery [6,7]. The core ATG proteins are subdivided into six functional modules based on physical interactions and their participation in different steps of autophagosome formation: the Atg1/ULK kinase complex, the Atg9/ATG9A-containing vesicles, the autophagy-specific phosphatidylinositol 3-kinase (PI3K) complex, the Atg2/ATG2–Atg18/WIPI complex, and the Atg8/LC3 and Atg12/ATG12 conjugation systems [6,7].

## Autophagosome formation mechanism

### Initiation

Autophagosome biogenesis can be induced by a variety of signals, such as nutrient starvation (including nitrogen, carbon, glucose, amino acids or phosphate deprivation), noxious stressors (including high temperature, hypoxia, redox imbalance or high salt), dysfunctional proteins and protein complexes, superfluous or damaged organelles and invading pathogens.

**Figure 2.**
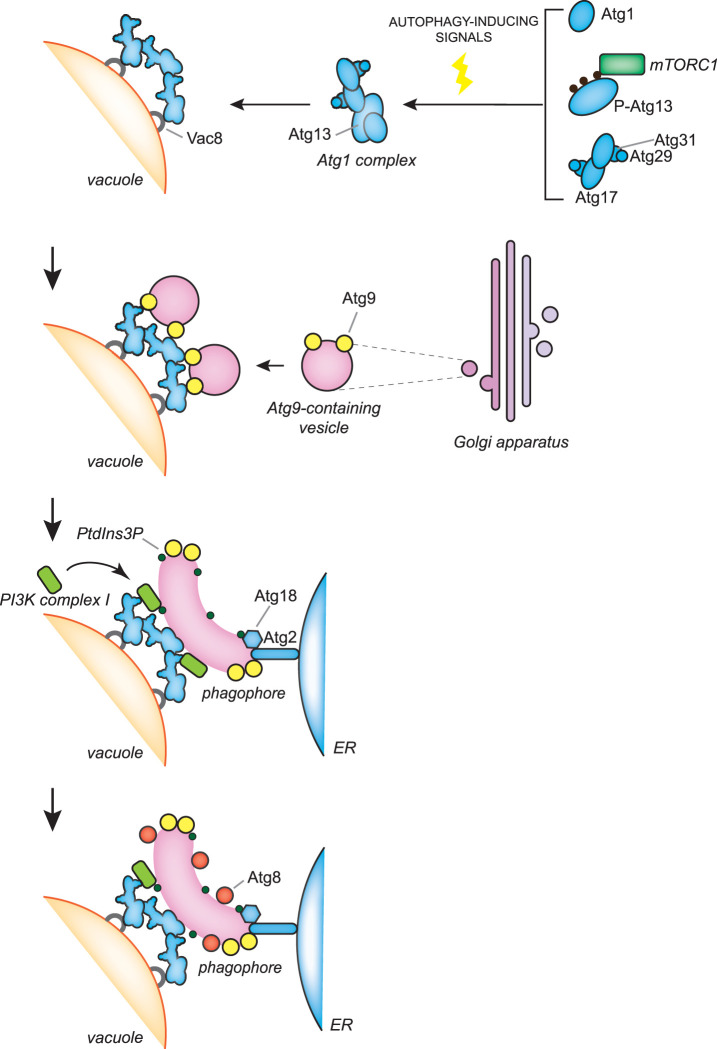
The molecular mechanisms of the early stages of autophagosome biogenesis during bulk autophagy in *S. cerevisiae* In *S. cerevisiae*, autophagosome biogenesis takes place at the PAS, which is adjacent to both the vacuole and ER. Autophagy induction by nutrient starvation leads to an inhibition of TORC1 activity and the concomitant dephosphorylation of Atg13 promotes the assembly of the Atg1 kinase complex, which comprises Atg1 and the Atg17–Atg31–Atg29 subcomplex. Atg1 kinase complexes assemble into a supra-structure, probably by liquid phase separation, which is anchored onto the vacuole via binding to Vac8. Atg9-containing vesicles, which are derived from the Golgi apparatus, are recruited onto those supra-structures and by possibly fusing together and with vesicles of other origins, generate the phagophore. The PI3K complex I is recruited to the PAS via an interaction with Vac8 and produces PtdIns3P on the phagophore membrane. This phosphoinositide is involved in the recruitment and assembly of the Atg2–Atg18 complex, which probably transfers lipids from the ER to the phagophore for its expansion. Atg2 binds Atg9 and PtdIns3P on the phagophore and one or more unknown proteins on the ER, while Atg18 associates to the phagophore by interacting with Atg2 and PtdIns3P. Another PtdIns3P-binding protein, Atg21, interacts with Atg16 to recruit the Atg12–Atg5–Atg16 complex, which is crucial for the conjugation of Atg8 to the PE present in the phagophore (see also Figure 4).

**Figure 3.**
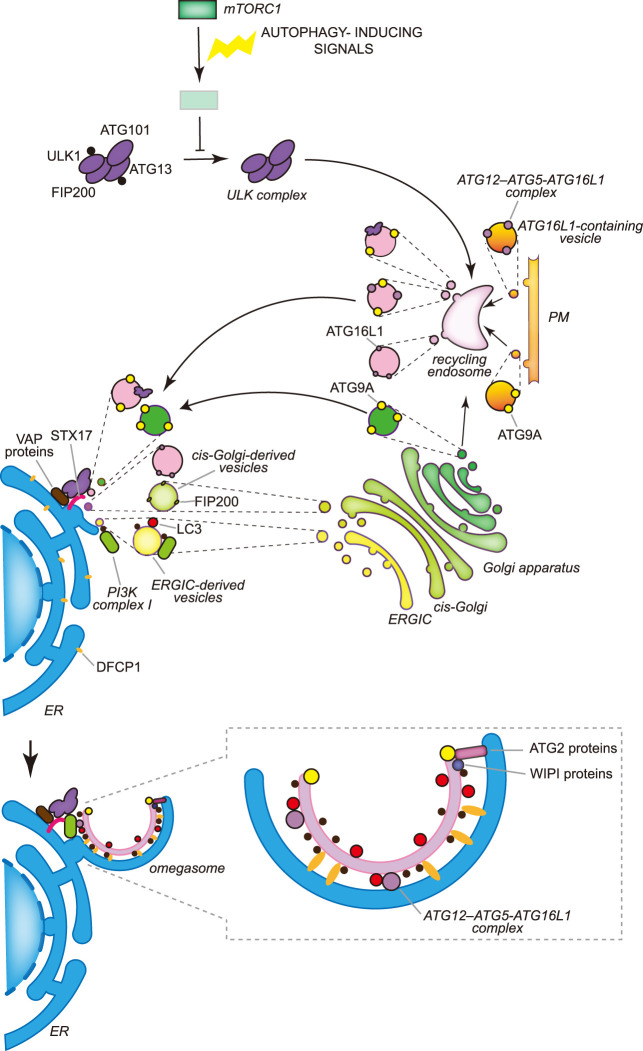
The molecular mechanisms of the early stages of autophagosome biogenesis during bulk autophagy in mammals. In mammalian cells multiple autophagosomes are simultaneously formed adjacently to the ER, in specific regions known as omegasomes, in which DFCP1 concentrates. The activity of the ULK kinase complex, which is constitutively formed by ULK1 or ULK2, ATG13, FIP200 and ATG101, is inhibited for example by mTORC1 in nutrient-rich conditions through the phosphorylation of ULK1 and ATG13. Nutrient deprivation leads to the inhibition of mTORC1 and the concomitant dephosphorylation of ULK1 and ATG13. Active ULK1 kinase complex associates to the recycling endosomes via an unknown mechanism and exits in vesicles that also contain ATG9A and/or the ATG12–ATG5–ATG16L1 complex. ATG9A reaches the recycling endosomes from either the Golgi apparatus or the plasma membrane (PM) by vesicular traffic, while ATG16L1 only from the PM. Recycling endosome-derived vesicles relocalize in the proximity of the ER, possibly through the binding of ULK1 and FIP200 with the ER transmembrane proteins VAPA and VAPB or STX17. The generation of the phagophore very likely involves the fusion of vesicles from different origins, including ATG9A-containing vesicles generated at the Golgi apparatus, COPII-coated vesicles derived from the ERGIC and the HyPAS. The HyPAS is a compartment formed through the fusion between ATG16L1-containing endosomal membranes and *cis*-Golgi-derived FIP200-positive vesicles, which is mediated by STX17 and its interactors SERCA2, E-SYT2 and SIGMAR1. The lipidation of LC3 already begins on these COPII-coated vesicles and relies on PtdIns3P, which is produced by the PI3K complex I. As a result, transport via COPII-coated vesicles is a possible mechanism for the localization of the PI3K complex I to the nascent phagophore. PtdIns3P is involved in the assembly of the complexes formed by ATG2 and WIPI proteins. The complexes formed by the ATG2 and WIPI proteins probably transfer lipids from the ER to the phagophore. Synthesis of PtdIns3P on the phagophore also leads to the association of ER-localized and PtdIns3P-binding DFCP1, which permits the generation of the characteristic omegasomes. Contrary to yeast, the distribution of the ATG proteins on the phagophore in mammalian cells is unknown and therefore what drawn in the figure is a speculative representation.

Several of these cues trigger autophagy via signaling cascades and many of them converge at the nutrient sensor target of rapamycin complex 1 (TORC1), to initiate bulk autophagy [8,9]. TORC1 modulates the activity of the Atg1/ULK kinase complex, the key molecular switch for autophagy, and possibly other components of the ATG machinery [8,9]. The Atg1/ULK kinase complex is composed of Atg1, Atg13, Atg17, Atg29 and Atg31 in yeast, and ULK1 or ULK2, ATG13, FIP200 and ATG101 in mammalian cells [8,9]. Although the Atg17–Atg31–Atg29 subcomplex is constitutively present at the PAS in yeast, the regulated association of Atg1 and Atg13 to this subcomplex and thus the formation of the Atg1 kinase complex requires TORC1 inactivation (Figure 2) [8,9]. Under nutrient-rich conditions, TORC1 phosphorylates Atg13 to block its interaction with Atg1 and the Atg17–Atg31–Atg29 subcomplex. Upon nutrient removal, TORC1 activity is inhibited and Atg13 is dephosphorylated. Dephosphorylated Atg13 binds to Atg1 and Atg17, leading to both the assembly of the Atg1 kinase complex and its kinase activity activation through Atg1 autophosphorylation (Figure 2). Unlike yeast, the mammalian ULK kinase complex is constitutively formed and mammalian TORC1 (mTORC1) inhibits the activity of this complex through the phosphorylation of ULK1 and ATG13 (Figure 3) [8,9]. In yeast, the cAMP-dependent protein kinase (PKA) can also negatively regulate autophagy via phosphorylation of Atg1 and Atg13, while the AMP-activated protein kinase (AMPK) can directly phosphorylate ULK1 to stimulate autophagy upon glucose starvation in mammalian cells [8].

In contrast with non-selective types, selective types of autophagy are induced by the cargo, which orchestrate the local biogenesis of an autophagosome around the targeted structure (Figure 1) [10]. Key players of this event are the autophagy receptors, which bind the structures to be degraded and tether them with the nascent phagophore [11,12]. While several autophagy receptors are associated with the cargo and are ubiquitin-independent, others recognize poly-ubiquitin chains that have been specifically conjugated on the surface of the targeted structures [10]. Extensively studied autophagy receptors are the soluble yeast Atg19 and mammalian p62/SQSTM1. While Atg19 specifically interacts with cytoplasmic oligomers principally formed by the precursor Ape1 protease [13], p62 can bind ubiquitin moieties linked for example to aggregates, mitochondria and bacteria [14]. Cargo-bound autophagy receptors then mediate the recruitment and activation of the Atg1/ULK kinase complex by interacting with yeast Atg11 or mammalian FIP200 [10,15,16]. This event, but probably also binding to the Atg8/LC3 proteins [14], initiates the local formation of an autophagosome (Figure 1).

### Membrane nucleation

Upon autophagy induction, the nucleation of the phagophore takes place at distinct subcellular places. In yeast, normally there is only one of these locations per cell and it is called the PAS [17–19] and it is found simultaneously adjacent to both the endoplasmic reticulum (ER) and vacuole [20,21]. The PAS is formed and tethered to the vacuole via binding between Vac8, a protein anchored into the vacuolar limiting membrane, and Atg13 (Figure 2) [22–24]. The mechanism underlying the connection between the PAS and the ER remains to be understood, but it may be mediated by the interaction between the subunits of the Atg1 kinase complex and various components of the ER exit sites (ERES), which are ER subdomains that are generally involved in the formation of COPII-coated vesicles [20,21,25]. In contrast, multiple phagophores are formed at the same time in the proximity of the ER in mammalian cells [26–28]. The recruitment of the ULK kinase complexes, possibly present on recycling endosome-derived vesicles and/or the hybrid pre-autophagosomal structure (HyPAS) (see below), onto the ER appears to involve binding of ULK1 and FIP200 with ER transmembrane proteins VAPA and VAPB [29], and ER-localized SNARE syntaxin 17 (STX17) (Figure 3) [30].

The supra-assembly of Atg1/ULK kinase complex, which has only been shown in yeast, is driven by a liquid phase separation mediated by the Atg13's intrinsically disordered region [31], and the resulting aggregate serves as a scaffold for the subsequent association and orchestration through phosphorylation of the function of the downstream ATG proteins [8,32,33]. In yeast, vesicles containing Atg9, the only transmembrane protein between the core ATG proteins, are recruited by the Atg1 kinase complex via the interaction with either Atg17 and the HORMA domain of Atg13 during bulk autophagy, or via Atg11 in selective types of autophagy, to possibly provide the seed membrane for the phagophore nucleation [34–37]. The Atg9-containing vesicles are clusters of tubules and vesicles, which are derived from the Golgi and move *en bloc* to provide at least some of the initial membranes of the PAS/phagophore (Figure 2) [38,39]. In addition, COPII-coated vesicles have also been shown to deliver membranes to the PAS and/or phagophore in yeast [40]; however, it remains unclear at which step of the autophagosome biogenesis this contribution is important. In mammalian cells, ATG9A-containing vesicles are derived from the *trans*-Golgi network and can traffic through the plasma membrane (PM) (Figure 3) [41,42]. Upon autophagy, ATG16L1-positive vesicles derived from the PM [43] and ATG9A-positive vesicles coalesce at the recycling endosome [42,44]. The ULK1-positive membranes exiting the recycling endosomes appear to reposition near the ER, possibly by interaction with ATG14, in a region that will become the omegasome (Figure 3) [45,46]. These membranes may serve as the seed for phagophore formation (see below) [42]. Moreover, the ER-to-Golgi intermediate compartment (ERGIC) is also a membrane source for autophagosomes, possibly by supplying membrane for the nucleation of the phagophore, a notion that is supported by the observation that ERES undergo a remodeling and associate with ERGIC upon nutrient starvation (Figure 3) [47]. Recently, it has been shown that *cis-*Golgi-derived FIP200-positive vesicles and ATG16L1-containing endosomal membranes fuse through a mechanism that depends on STX17, the SERCA2 calcium pump, the calcium-regulated membrane tether E-SYT2 and the ER-localized transmembrane calcium regulator SIGMAR1 [48]. The resulting compartment is a HyPAS, which is important for autophagy initiation and appears to be key in the recognition of diverse cargoes during selective types of autophagy [48]. It remains to be understood whether the *cis-*Golgi-derived FIP200-positive vesicles are in fact the ERGIC-derived vesicles, and whether the HyPAS may correspond to the recycling endosome-derived vesicles that were found to be positive for ATG16L1 and ULK1.

The autophagy-specific PI3K complex is known as complex I to distinguish it from the PI3K complex II, which is mostly involved in regulating endosomal traffic and functions [49,50]. The PI3K complex I is composed of the class III PI3K Vps34, Vps15, Vps30/Atg6, Atg14 and Atg38 in yeast, and VPS34, p150, BECN1, ATG14L and NRBF2 in mammalian cells [49–52], and its activity is enhanced upon phosphorylation by the Atg1/ULK kinase complex of some of its subunits [8]. The phosphatidylinositol 3-phosphate (PtdIns3P) synthesized by the PI3K complex I plays a key role in the recruitment and assembly of the rest of the ATG machinery (see below) [19]. In mammalian cells, the generation of PtdIns3P leads to the recruitment of the ER integral membrane protein DFCP1 that specifically binds this lipid, creating a close association between the external surface of expanding phagophore and the ER [53–55]. These characteristic ER subdomains are known as omegasomes [53]. The PI3K complex I appears to be recruited at the site of phagophore nucleation after the arrival of the Atg9/ATG9A-containing vesicles, by also interacting with Vac8/ARMC3, at least in yeast and specific tissues in mammals [19,28,56]. Furthermore, there may be two additional mechanisms for the recruitment of the PI3K complex I that are not mutually exclusive. The first is that the PI3K complex I may be transported via vesicles derived from ERGIC, to which the COPII-coated vesicles are recruited in a PI3K complex-dependent manner under autophagy-inducing conditions (Figure 3) [57]. In the second, ATG14L (and by the extension the PI3K complex I) may be recruited to the ER or eventually the HyPAS, by interacting with STX17 (Figure 3) [58].

### Membrane expansion

The following step in the autophagosome biogenesis is the expansion of the phagophore, which requires an enormous supply of lipids and the function of multiple core ATG proteins. The PtdIns3P present on the phagophore is particularly important for the recruitment of ATG proteins that mediates its expansion [6,7]. Central effectors of this phosphoinositide are the members of the PtdIns3P-binding proteins from the WD-repeat protein interacting with phosphoinositides (WIPI) protein family, yeast Atg18 and Atg21, and mammalian WIPI1 to WIPI4 [59]. These beta-propeller proteins directly bind PtdIns3P and interact with the functional modules crucial for the phagophore expansion: the Atg2/ATG2–Atg18/WIPI complex, and two interconnected Atg8/LC3 and Atg12/ATG12 conjugation systems [59,60].

In yeast, the Atg2–Atg18 complex assembles sequentially at the membrane contact sites (MCSs) between the ER and the extremities of the phagophore [20,21,61]. Its recruitment at this peculiar location depends on its interaction with Atg9 and PtdIns3P (Figure 2) [61]. In mammalian cells, TRAPPC11, a subunit of the human transport protein particle (TRAPP) III complex [62], is involved in the recruitment of the complex between ATG2 proteins and WIPI4 (Figure 3) [63]. *In vitro* experiments have shown that both yeast Atg2 and mammalian ATG2 proteins have membrane tethering and lipid transfer functions [64–66]. While the C-terminal part of the Atg2/ATG2 proteins binds to the phagophore in cooperation with Atg9/ATG9A, Atg18/WIPI4 and PtdIns3P [61,64,65,67,68], the N-terminus of the ATG2 proteins interacts with the ER and capture lipids that are transferred to the phagophore through the long hydrophobic groove in their middle (Figures 2 and 3) [64,65,68]. These lipids accumulate on the cytoplasmic leaflet of the phagophore membrane and must be flipped to the luminal leaflets to promote phagophore expansion. Atg9/ATG9A forms homotrimers, which contain lateral and vertical cavities, and through a still obscure molecular mechanism scrambles lipids unevenly distributed between the two lipid bilayers of the phagophore membrane, promoting phagophore expansion (Figures 2 and 3) [69,70].

**Figure 4.**
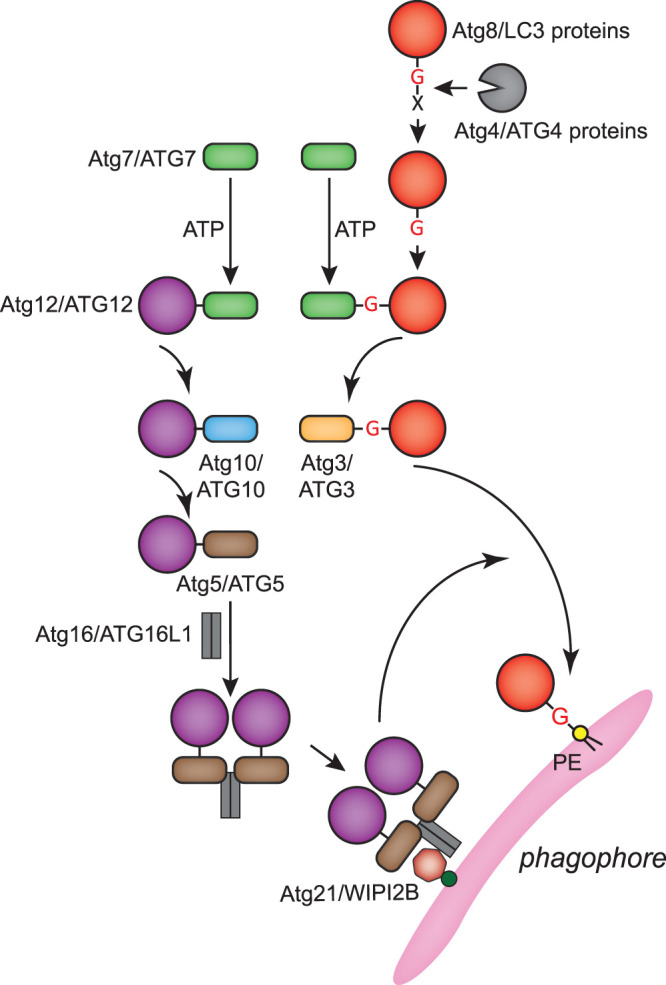
Atg8/LC3 and Atg12/ATG12 conjugation systems. Atg12/ATG12 and Atg8/LC3 proteins are ubiquitin-like proteins. The conjugation systems of these two proteins are interconnected and they ultimately covalently link Atg8/LC3 proteins to the PE present in the phagophore membrane during autophagy. Amino acids at the C-terminus of Atg8/LC3 proteins (indicated with a X in the draw) are post-translationally cleaved by the Atg4/ATG4 proteases to expose a C-terminal glycine (G). Processed Atg8/LC3 proteins are activated and finally conjugated to the amino group of PE through the sequential action of the E1 enzyme Atg7/ATG7 and the E2 enzyme Atg3/ATG3. The last step of this reaction is mediated by the Atg12–Atg5–Atg16/ATG12–ATG5–ATG16L1 complex, which is formed by the Atg12/ATG12 conjugation system and acts as an E3 enzyme. The Atg12/ATG12 conjugation system involves the sequential action of Atg7/ATG7 and the E2 enzyme Atg10/ATG10, and covalently links Atg12/ATG12 to Atg5/ATG5. The Atg12/ATG12–Atg5/ATG5 conjugate binds to Atg16/ATG16L1, which subsequently multimerizes to form the Atg12–Atg5–Atg16/ATG12–ATG5–ATG16L1 complex. This complex associates with the phagophore via interaction with both Atg21/WIPI2B and PtdIns3P.

The two interconnected ubiquitin-like conjugation systems drive cargo sequestration, membrane expansion and autophagosome completion. Their action leads to the conjugation of the members of the Atg8/LC3 ubiquitin-like protein family to the phosphatidylethanolamine (PE) present in the phagophore membrane (Figure 4) [71]. While yeast possesses one Atg8 protein, mammalian cells have six main isoforms of Atg8-like proteins, i.e. three LC3 paralogs (LC3A, LC3B and LC3C) and three GABARAP paralogs (GABARAP, GABARAP-L1 and GABARAP-L2) [71]. Atg8/LC3 proteins are post-translationally processed by the Atg4/ATG4 proteases to expose a C-terminal glycine. Upon autophagy induction, this glycine is used for the covalent linkage to the amino group of PE through the sequential action of the E1 enzyme Atg7/ATG7, the E2 enzyme Atg3/ATG3 and the E3 enzyme activity of the Atg12–Atg5–Atg16/ATG12–ATG5–ATG16L1 complex (Figure 4) [71]. Atg12/ATG12 is also an ubiquitin-like protein that is covalently conjugated to Atg5/ATG5 through a catalysis mediated by Atg7/ATG7 and the E2 enzyme Atg10/ATG10 (Figure 4) [71]. The Atg12–Atg5/ATG12–ATG5 conjugate subsequently binds with Atg16/ATG16L1 and homo-oligomerizes [71]. The resulting complex associates to the phagophore membrane by interacting with Atg21/WIPI2B (Figure 4) [72,73]. Of note, Atg8/LC3 proteins can also be conjugated to membranes other than the ones of phagophores, where they carry out non-autophagy related functions [74].

PE-conjugated Atg8/LC3 proteins have a preferred orientation towards the membrane, which exposes their two hydrophobic pockets to recognize proteins that possess Atg8 protein-interacting motif (AIM)/LC3-interacting region (LIR), such as autophagy receptors and cargoes in selective types of autophagy [14]. Although there is no conclusion yet on how PE-conjugated Atg8/LC3 proteins regulate autophagosome biogenesis, their tether and/or hemifusion properties might be involved in phagophore membrane expansion [75]. In fact, Atg8/LC3 conjugation to PE leads to a deformation of giant unilamellar vesicles (GUVs) by inducing the interaction between two aromatic residues at the C-terminus of Atg8/LC3 proteins and the outer lipid layer of the GUVs [76]. These two aromatic residues are important for the autophagosome biogenesis during bulk autophagy in yeast and the degradation efficiency of a major selective cargo p62 for autophagy in mammals [76]. These properties may also contribute to the formation of a proteinaceous coat by Atg8-PE [77]. PE-conjugated Atg8/LC3 proteins could also indirectly modulate phagophore growth through the recruitment of other proteins. For example, both Atg1 and ULK1 are recruited to the convex membrane of the phagophore through binding to Atg8/LC3 proteins [78,79].

Membrane supply is a key factor for phagophore expansion and in addition to capturing lipids from ER through Atg2/ATG2 proteins (Figures 2 and 3), other mechanisms may exist. For example, ultrastructural investigations have revealed that mammalian phagophores may have additional MCSs [54,55,80]. The mammalian autophagosomes biogenesis occurs at the ER subdomain enriched in both the ER membrane VMP1 and TMEM41B, which modulates ER-phagophore MCSs [28,80,81], and the phosphatidylinositol synthase (PIS) [46]. Moreover, *de novo* lipid synthesis is also a possible lipid source for phagophore expansion. A recent study in yeast has revealed that the acyl-CoA synthetase Faa1, which is also localized on the phagophore, activates fatty acids to convey them into the synthesis of phospholipids at the phagophore-ERES MCSs and promotes their incorporation into Atg8-positive membranes [82]. Mitochondria and lipid droplets are also possible lipid sources for phagophore expansion, but their contribution is probably indirect [7].

### Membrane shaping

The curvature of the phagophore membrane during its expansion is pivotal for autophagosome biogenesis, especially during bulk autophagy because this event is not guided by the cargo. Proteins sensing and generating membrane curvature, and the physical properties of the membrane appear to be involved in this process.

The yeast Atg12–Atg5–Atg16 complex binds membranes and assembles into a mesh-like structure in association with Atg8 lipidation on artificial vesicles [77,83], which might serve as a scaffold to shape the phagophore. While the copy number of Atg12–Atg5–Atg16 complexes present at the PAS is too small to cover the entire surface of an autophagosome with an average size [84], it is possible that these proteins affect the shaping of the phagophore. This latter notion is supported by the observation that Atg8-PE can also shape the membrane of synthetic vesicles (Figure 2) [76]. Consistently, blocking the conjugation of Atg8/LC3 proteins leads to the accumulation of phagophore with abnormal morphology in mammalian cells [85–88]. Finally, Atg8/LC3 proteins tend to be lipidated and sorted to membrane regions with high curvature, which probably enhances the generation of curved membrane and/or stabilizes the membrane curvature [76,89,90].

Actin filaments do not only recruit ATG proteins in the early stage of autophagosome biogenesis [91], but they also contribute to the shaping of the phagophore membrane in mammalian cells [92]. Actin filaments are depolymerized shortly after nutrient starvation and actin is re-assembled into an intricate network on the concave side of the phagophore through a mechanism that depends on the actin-branching Arp2/3 complex [92]. In this process, the actin-capping protein CapZ stimulates actin polymerization by binding to PtdIns3P [92]. The resulting actin filaments serve as a cytoskeletal scaffold to develop phagophores into autophagosomes with normal morphology. It has been biophysically shown that the highly curved edges of disc-like membranous vesicles, like phagophores, are energetically unstable and this can be a factor that affects their shape [93]. With the disc expanding, the areas at the edges increase and the disc becomes more unstable [93], which could be compensated by the spontaneous acquisition of a spherical structure. Based on this model, the physical properties of the lipid bilayer can also be an important factor for the shaping of the phagophore. To generate autophagosomes with normal sizes, it is important to delay the spontaneous spherical rearrangement of the expanding phagophore. There are two main hypotheses linked to this concept [6,94]. One is that the highly curved edge of the phagophore is stabilized by membrane-binding proteins that are asymmetrically distributed on the inner and outer membrane of the phagophore [94,95]. The lipidated Atg8/LC3 proteins on the phagophore membrane could be an edge-stabilizing candidate and in line with this notion, it is known that their amount correlates with the size of autophagosomes [75,96,97]. The other hypothesis is that an alteration of the lipid composition of the phagophore membrane [94]. For example, the phagophore fuses with small vesicles such as the Atg9/ATG9A-containing vesicles or the COPII-coated vesicles, which have higher membrane to lumen ratio that can reduce the distance between the two parallel bilayers of the growing phagophore. This will in turn increase the curvature at the edges of the membrane and consequently accelerate the bending of the phagophore.

### Pore closure and autophagosome maturation

The phagophore expansion and its bending into a spherical shape lead to a close proximity of its extremities, and the closure of the remaining pore is the last step for the autophagosome formation (Figure 1). This process involves a membrane fission event, which is mediated by the endosomal sorting complexes required for transport (ESCRT) machinery in both yeast and mammalian cells [98–101]. However, this result remains contradictory since in the absence of Vps4, a component of ESCRT machinery, autophagy progresses normally in yeast [102]. It has also been reported that the Rab5-like GTPase Vps21 and Atg14 are involved in the pore closure [98], but this remains to be fully understood.

Upon completion, autophagosomes undergo a maturation that appears to be concomitant with their detachment from the ER [27,53]. Autophagosome maturation is characterized by the release of most of the ATG proteins from their surface into the cytoplasm for reuse, which also involves the turnover of PtdIns3P by phosphatases from the myotubularin family [103–105]. One exception is the Atg8/LC3 proteins, which are present on both the inner and outer side of the phagophore membrane. The ones on the surface of complete autophagosomes are released through cleavage by the Atg4/ATG4 proteases [71], whose proteolytic function is blocked by the phosphorylation of Atg1/ULK1 during the phagophore expansion [106,107]. This post-translational modification inhibits the deconjugation of Atg8/LC3 proteins from the PE and ensures the expansion of the phagophore [106,107]. In addition, part of Atg1/ULK1 also remains associated with the autophagosome by interaction Atg8/LC3 proteins, and this pool of Atg1/ULK1 may facilitate autophagosome maturation and/or its fusion with vacuoles/lysosomes [108]. Atg9 is also present on the surface of the yeast autophagosomes [39,103], but the functional relevance of this remains to be uncovered.

### Fusion

Autophagosomes form adjacently to the vacuole in yeast, whereas they are transported in close proximity of vacuoles and late endosomes/lysosomes in plant and mammalian cells, through a process that requires microtubules and related motor proteins [109]. The transport of autophagosomes to lysosomes also depends on the WASP homolog associated with actin, membranes, and microtubules (WHAMM) complex, which drives this motility through the Arp2/3 complex-dependent actin comet tail mechanism [110]. Fusion of autophagosomes with degradative organelles begins with tethering, which involves the homotypic vacuole fusion and protein sorting (HOPS) tethering complex [111]. Tethering leads to the formation of specific complexes between autophagosomal SNAREs such as yeast Ykt6, mammalian STX17 and YKT6 and vacuolar/lysosomal SNAREs like yeast Vam3, Vam7 and Vti1, and mammalian VAMP7 and VAMP8, which ultimately drive the fusion between autophagosomes and vacuoles, late endosomes or lysosomes [111]. Finally, the inner membrane of autophagosomes and sequestered cargoes are degraded in by resident vacuolar/lysosomal hydrolases and the resulting building blocks are transported back to the cytoplasm for recycling [1,6]. The fusion of autophagosomes with the compartments of the endolysosomal system has been covered in details by recent reviews [111–114].

## Perspectives

- *Importance of the field.* Autophagy is essential to maintain cellular homeostasis in countless situations. Unveiling the mechanism of this process is critical to understand the pathophysiology of autophagy-related diseases and might provide insight into therapeutic interventions. ATG proteins play a central role in autophagosome biogenesis and their functions have been gradually elucidated during last few decades, providing increasing insights into the molecular mechanism of autophagy.
- *Summary of the current thinking.* The signal cascades triggering autophagy and how these orchestrate ATG proteins are becoming better understood. While the exact membrane contribution of each source for autophagosome biogenesis is still largely unknown, studies have revealed that Atg9/ATG9A-containing, ATG16L1-positive and COPII-coated vesicles are important in supplying key proteins and lipids to the PAS/phagophore, while the ER is an essential membrane source for phagophore expansion. Moreover, increasing evidence suggests that the shaping of the phagophore membrane depends not only on proteins sensing and generating membrane curvature, but probably also on biophysical properties of the membrane.
- *Future directions.* There are still several central questions that remain to be answered. For example, it is largely unclear how the phagophore-ER MCSs are generated, especially *in vivo*. Moreover, it is unknown the mechanism underlying the unidirectional flux of lipids from the ER to the phagophore, since the Atg2/ATG2 proteins as well as the scramblases activities in the phagophore and eventually in the ER (i.e.VMP1 and TMEM41B) [115], probably do not produce the energy necessary to catalyse lipid transfer. Addressing these questions but also other ones, will help to increase our knowledge and shed new light into the molecular mechanism of autophagy.

## Open Access

Open access for this article was enabled by the participation of University of Groningen in an all-inclusive *Read & Publish* pilot with Portland Press and the Biochemical Society.

## Abbreviations

AIM: Atg8 protein-interacting motif
ERES: ER exit sites
ERGIC: ER-to-Golgi intermediate compartment
ESCRT: endosomal sorting complexes required for transport
GUVs: giant unilamellar vesicles
HyPAS: hybrid pre-autophagosomal structure
LIR: LC3-interacting region
mTORC1: mammalian TORC1
PAS: phagophore assembly sites
PE: phosphatidylethanolamine
PI3K: phosphatidylinositol 3-kinase
TRAPP: transport protein particle
